# *In silico* identification of conserved *cis*-acting RNA elements in the SARS-CoV-2 genome

**DOI:** 10.2217/fvl-2020-0163

**Published:** 2020-07-29

**Authors:** Bader Y Alhatlani

**Affiliations:** 1 Department of Medical Applied Sciences, Unayzah Community College, Qassim University, Unayzah, Saudi Arabia

**Keywords:** *cis*-acting RNA elements, RNA structure, RNA virus, SARS-CoV-2

## Abstract

**Aim:**

The aim of this study was to computationally predict conserved RNA sequences and structures known as *cis*-acting RNA elements (CREs) in the severe acute respiratory syndrome coronavirus 2 (SARS-CoV-2) genome.

**Materials & methods:**

Bioinformatics tools were used to analyze and predict CREs by obtaining viral sequences from available databases.

**Results:**

Computational analysis revealed the presence of RNA stem-loop structures within the 3′ end of the ORF1ab region analogous to previously identified SARS-CoV genomic packaging signals. Alignment-based RNA secondary structure predictions of the 5′ end of the SARS-CoV-2 genome also identified conserved CREs.

**Conclusion:**

These CREs may be potential vaccine and/or antiviral therapeutic targets; however, further studies are warranted to confirm their roles in the SARS-CoV-2 life cycle.

In December 2019, a novel coronavirus, initially named 2019-nCoV, was found to be the causative agent for an outbreak of pneumonia in patients that visited a wet market in Wuhan, China [1]. The virus was subsequently renamed severe acute respiratory syndrome coronavirus 2 (SARS-CoV-2) and identified to be from the *Betacoronavirus* genus [2]. SARS-CoV-2 is responsible for the ongoing global pandemic of coronavirus disease 2019, which as of 22 May 2020 has caused more than 5 million confirmed cases and more than 332,000 deaths worldwide according to the WHO. Although there have been great efforts to develop an effective vaccine or specific antiviral treatment, neither is available yet. The Coronaviridae family contains a variety of viruses that cause a wide range of diseases, including respiratory, enteric, hepatic and neurological diseases in human and animals [3]. Most of the human coronaviruses usually cause mild symptoms; however, two human coronaviruses, severe acute respiratory syndrome (SARS-CoV) and Middle East respiratory syndrome (MERS-CoV), were identified to be highly pathogenic in humans [4,5]. Coronaviruses (CoVs) are generally grouped into four genera: *Alphacoronavirus*, *Betacoronavirus*, *Gammacoronavirus* and *Deltacoronavirus* [6,7].

CoVs have the largest known genomes among RNA viruses, which range from about 26 to 32 kb in length and contain an enveloped positive-sense, single-stranded RNA molecule (+ve ssRNA) that is capped at the 5′ end and polyadenylated at the 3′ end [8]. SARS-CoV2 shares genomic features with other SARS-like CoVs with complete genomic similarities of about 88% to bat SARS-like CoVs and 79% to SARS-CoV [9]. The SARS-CoV-2 genome is organized into about 13 open reading frames (ORFs) and two-thirds of the genome is occupied by the 5′-terminal region overlapping ORF1a and ORF1b, which are translated from the genomic RNA to encode the replicase polyproteins pp1a and pp1b [9,10].

Like most RNA viruses, the genomes of CoVs contain *cis*-acting RNA elements (CREs) and stem-loop structures that interact with RNA and viral or host proteins to form RNA–RNA or RNA–protein interactions to facilitate viral replication, translation and genome packaging [11,12]. While often these CREs are located at the 5′ and 3′ untranslated regions (UTRs), they can also be found within the coding regions of CoV genomes [13–15]. These important regions of the viral genome may be potential targets for SARS-CoV-2 antiviral therapeutics. Therefore, the aim of this study was to locate *cis*-acting regulatory elements within the SARS-CoV-2 genome using bioinformatics approach.

## Materials & methods

### GenBank accession numbers of viral sequences

Viral genomic sequences were retrieved from GenBank (National Center for Biotechnology Information [NCBI]). Virus strains and accession IDs used in this study were: (NC_045512.2) for SARS-CoV-2, (NC_004718.3) for SARS-CoV and (MG772933) for bat SARS-like CoV.

### Bioinformatics analysis

The RNA secondary structures of the viral genomic sequences were predicted using the online Mfold web server at http://unafold.rna.albany.edu/?q=mfold/ and RNAfold web server at http://rna.tbi.univie.ac.at/cgi-bin/RNAWebSuite/RNAfold.cgi [16,17]. In addition, the LocARNA web server at http://rna.informatik.uni-freiburg.de/LocARNA/Input.jsp was used for the alignment-based prediction of consensus RNA secondary structures at the 5′-terminal region of SARS-CoV-2, bat SARS-like CoV and SARS-CoV genomes [18]. The VARNA web applet at http://varna.lri.fr/ was then used to draw RNA secondary structures [19].

## Results

Previous studies on betacoronaviruses such as mouse hepatitis coronavirus (MHV), MERS-CoV and SARS-CoV revealed that the ORF1b region may contain CREs suggested to function as packaging signals (PSs) [20,21]. These CREs are functionally and structurally conserved within the same lineages of betacoronaviruses [14]. A previous bioinformatics study on the SARS-CoV genome predicted a stable stem-loop RNA structure at the 3′ end of ORF1b, encompassing nucleotides (nts) 19888–19950, to be the putative core PS (PS_core_) of SARS-CoV [22]. A further functional analysis identified the PS of the SARS-CoV as a 580 nt sequence encompassing viral genomic RNA (nt range: 19712–20294) that fold into a RNA secondary structure, including the PS_core_ and binds to the nucleocapsid (N) protein [21]. SARS-CoV-2 shares 87.6 and 79% complete genome similarities with bat SARS-like CoV and SARS-CoV, respectively [9,23].

**Figure 1.**
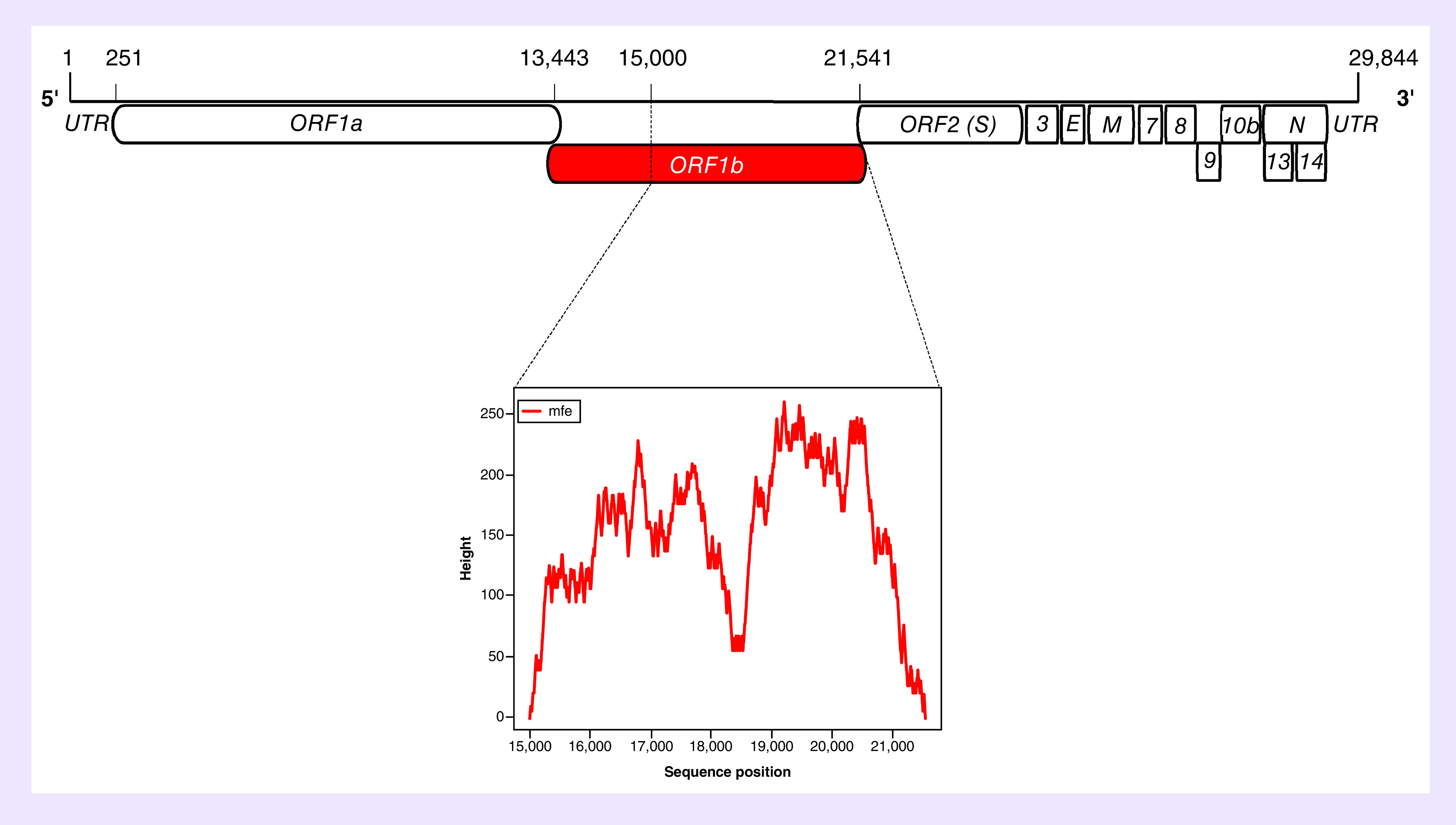
Prediction of minimum free energy structures within the severe acute respiratory syndrome coronavirus 2 ORF1b region. The SARS-CoV-2 genome is about 30 kb in length and organized in 13 ORFs. The viral genome is flanked by the 5′ and 3′ UTRs. The ORF1b region is highlighted in red (upper panel) and a mounting plot represents the prediction of the MFE structures spanning from nt position 15,000–21,541 of the SARS-CoV-2 genome sequences (NC_045512.2) is shown with the highest peak of MFE prediction located between 19,000 and about 20,300 nt of the SARS-CoV-2 ORF1b region (lower panel). Note that the mountain plot indicates secondary structure in a plot of height versus sequence position, where the peaks represent the probabilities of RNA base pairing. MFE: Minimum free energy; nt: Nucleotide; ORF: Open reading frame; SARS-CoV-2: Severe acute respiratory syndrome coronavirus 2; UTR: Untranslated region.

**Figure 2.**
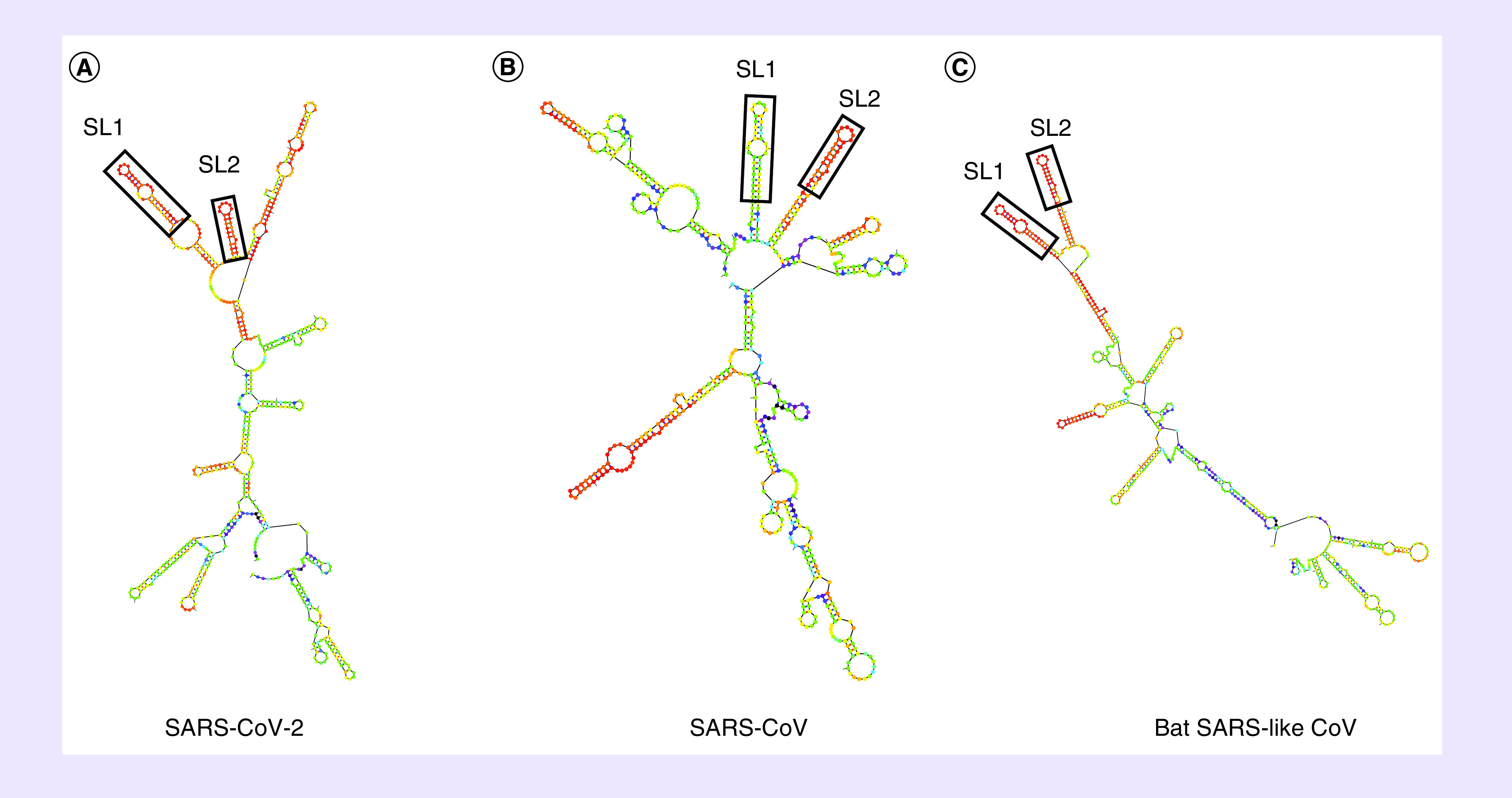
Prediction of ORF1b RNA secondary structures. RNA secondary structures located at the 3′ end of the SARS-CoV-2 ORF1b region (nucleotide positions: 19,712–20,294) were predicted using the Mfold web server **(A)** and compared with RNA secondary structures at the same position for SARS-CoV (NC_004718.3) **(B)** and bat SARS-like CoV (MG772933) **(C)**. The two identical RNA stem-loops (SL1 and SL2) are indicated in a rectangle among the three viruses. SARS-CoV-2: Severe acute respiratory syndrome coronavirus 2.

**Figure 3.**
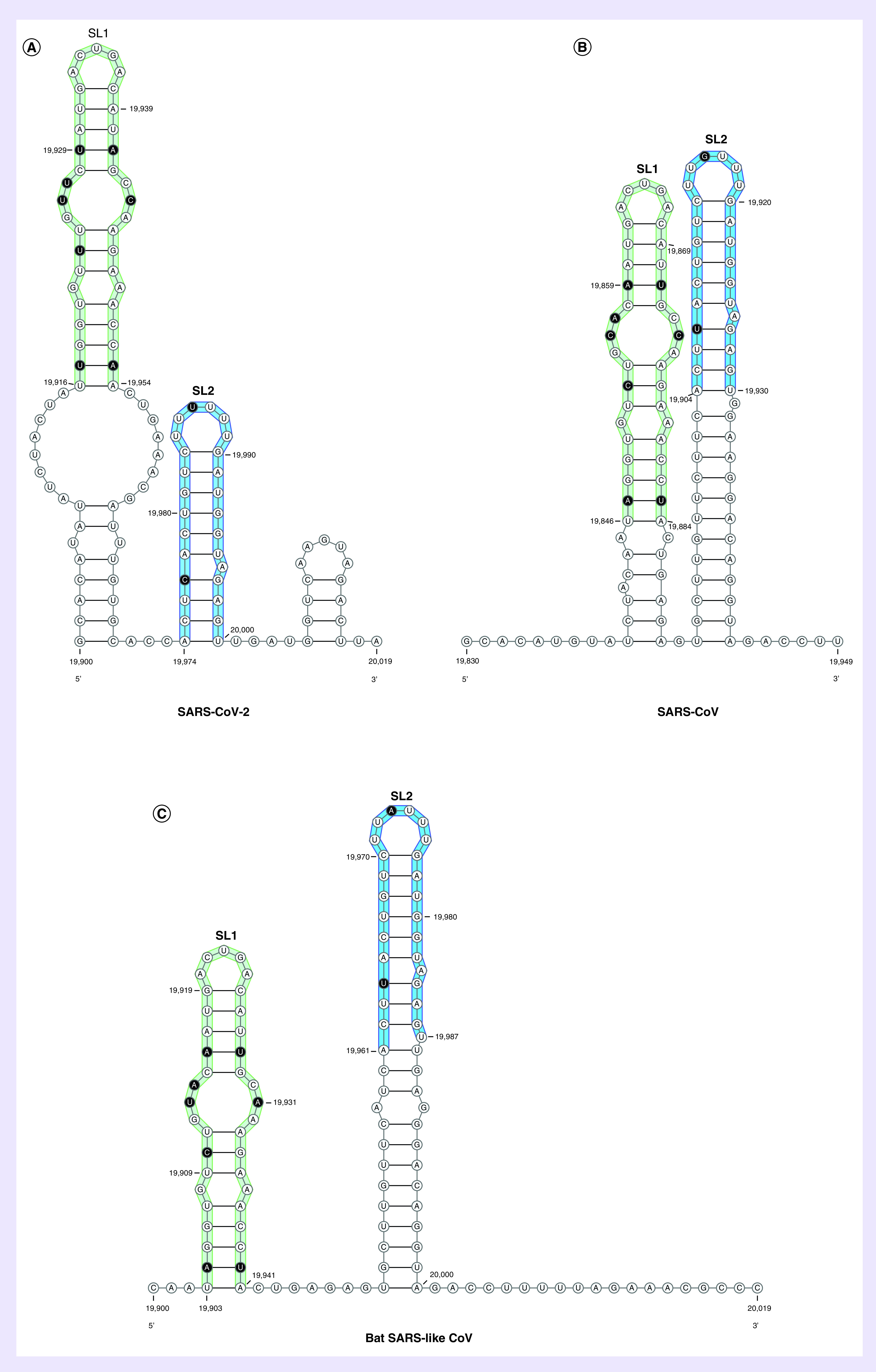
Comparison of the predicted RNA secondary structures among Severe Acute Respiratory Syndrome Coronavirus 2. The viral RNA sequences in the 3′ end of the ORF1b region from 19,900 to 20,019 nucleotide for both SARS-CoV-2 and bat SARS-like CoV and in the region of 19,830–19,949 nucleotide for SARS-CoV, were folded using the Mfold web server and drawn using the VARNA web applet to compare predicted RNA stem-loops. The similarities of the RNA structures of the predicted SL1 and SL2 among the three viruses are highlighted in green and blue, respectively. In addition, the sequence variation in these two stem-loops are indicated in black. These two RNA stem-loops are part of the previously identified SARS-CoV genomic PS, with SL2 being identified as the PS_core_ [21,22]. SARS-CoV-2: Severe acute respiratory syndrome coronavirus 2.

Hence, in this study, bioinformatics analysis was used to identify if the ORF1b region of SARS-CoV-2 possesses *cis*-acting RNA elements similar to that observed in SARS-CoV and other CoVs. To test this hypothesis, the SARS-CoV-2 ORF1b region was analyzed for RNA secondary structures and compared with the closely related SARS-CoV and bat SARS-like CoV sequences. SARS-CoV-2 RNA sequences spanning nts 15,000–21,541 were first analyzed using the RNAfold web server to predict the minimum free energy [17]. Results predicted the lowest free energy was located between nts 19,000 and 20,300 of the SARS-CoV-2 ORF1b region (Figure 1). Because this position overlaps with the genomic PS of SARS-CoV, which was identified at nt positions 19,712–20,294, the Mfold web server was then used to predict the RNA secondary structures spanning nts 19,712–20,294 of the SARS-CoV-2 ORF1b region and were compared with those of SARS-CoV and bat SARS-like CoV [16]. The Mfold analysis resulted in differences in the predicted RNA secondary structures between the three sequences; however, two stable stem-loops were identified to be similar and observed in all three viruses (Figure 2A–C). The predicted two stem-loops (named as SL1 and SL2) were located at nt positions 19,900–20,000, 19,839–19,943 and 19,903–20,000 of the SARS-CoV-2, SARS-CoV and bat SARS-like CoV genomes, respectively (Figure 3A–C). The upper part of SL1, which contains 38 nts, is structurally conserved between the three viruses, with covariation in the sequences. In the case of SARS-CoV-2, the SL1 is 70 nts and longer than those predicted in SARS-CoV and bat SARS-like CoV, whereas its length in SARS-CoV is 51 nts and only the upper part of SL1 is formed in bat SARS-like CoV (Figure 3A–C). The SL2 is much shorter in SARS-CoV-2, with only 26 nts, compared with that predicted in SARS-CoV and bat SARS-like CoV, which is 51 nts in length for both viruses. However, the genetic sequences of the upper part of SL2 were more conserved among the three viruses than SL1, with only a two nucleotide difference among SARS-CoV-2, SARS-CoV and bat SARS-like CoV. The first covariant is located at the stem and includes a C-G base pair in the SARS-CoV-2 sequence in place of a U-G base pair found in SARS-CoV and bat SARS-like CoV (Figure 3A–C). This single nucleotide difference from a C to U maintains the base pairing of the stem and does not change the amino acid (Leu) of ORF1b (Figure 3A–C). The second covariant is located at the U-rich loop and it is the third base in the loop in which this nucleotide is a U in the SL2 of SARS-CoV-2. It is a G and an A in the SARS-CoV and bat SARS-like CoV sequences, respectively. However, this single nucleotide difference changed the amino acid from Leu to Phe at this position in the SARS-CoV-2 genome (Figure 3A–C). It should be noted that the SL2 that was the previously predicted to function as the putative core PS (PS_core_) of SARS-CoV genome [21,22]. Therefore, it is reasonable to assume that the predicted RNA stem-loop structures of SARS-CoV-2 may also have the same role as a putative genomic packaging signal, given the conservation of the RNA structures and SL1 and SL2 sequences and similarities of the genomic locations of these predicted RNA structures within ORF1b.

**Figure 4.**
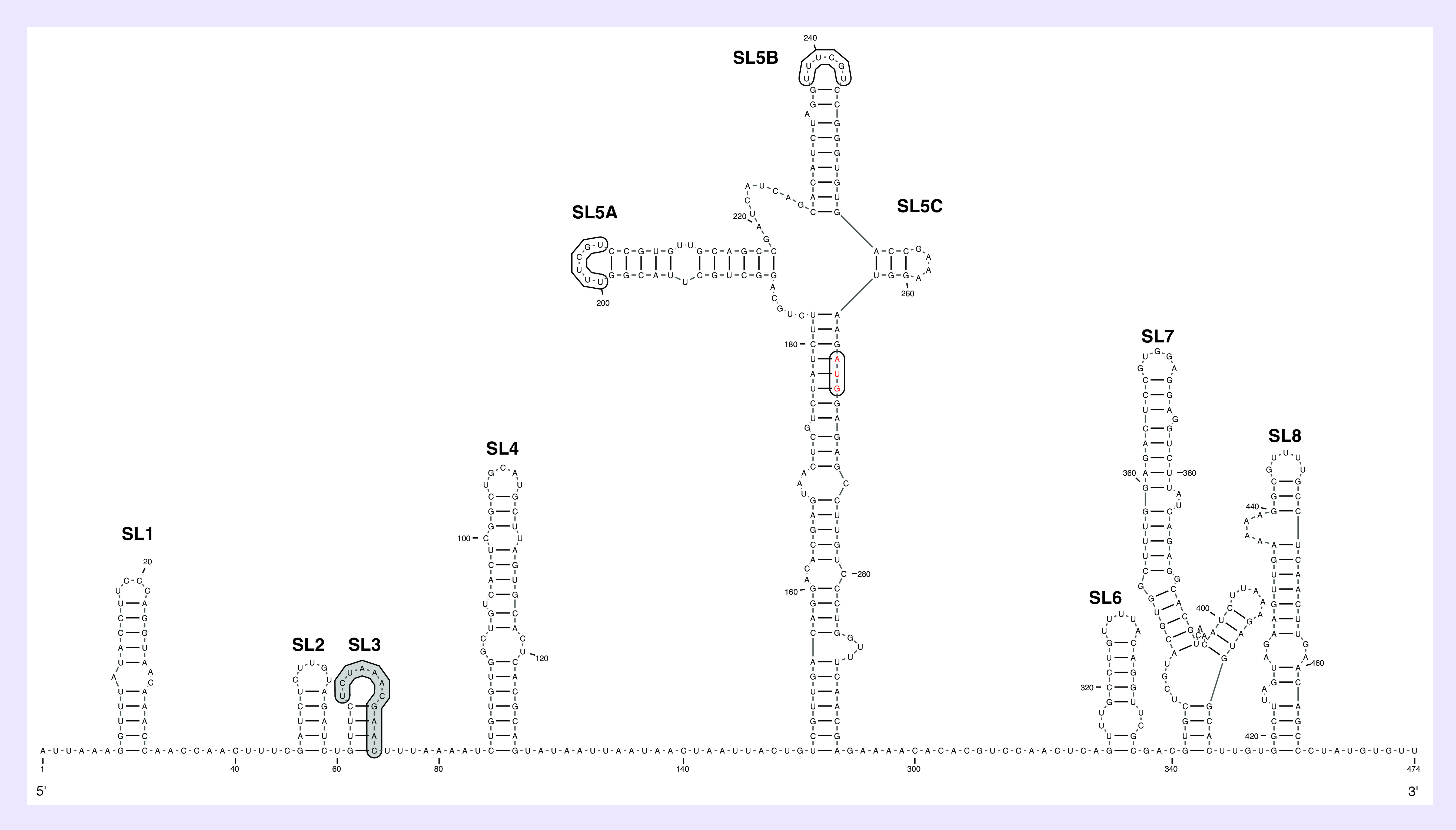
Computational prediction of RNA secondary structures model at the severe acute respiratory syndrome coronavirus 2 5′-terminal region. Schematic representation of the RNA secondary structures at the 5′ region of the severe acute respiratory syndrome coronavirus 2 (SARS-CoV-2) genome (nucleotide positions: 1–474) was generated using the Mfold web server and drawn using the VARNA web applet (ΔG = -155.50 kcal/mol). The eight RNA stem-loops (SL1–SL8) are indicated and the conserved core leader transcriptional regulatory sequence (TRS) region located within the SL3 is highlighted in gray. In addition, the conserved 5′-UUUCGU-3′ motifs found within SL5A and SL5B are indicated in boxes and the AUG start codon of ORF1ab is indicated in red. The SARS-CoV-2 reference genome sequence obtained from GenBank (NC_045512.2) was used.

*Cis*-acting RNA element secondary structures and sequences have also been previously described at the 5′ end of a number of CoVs, including MHV, bovine coronavirus (BCoV), MERS-CoV and SARS-CoV [15]. RNA secondary structures at the 5′ region of the SARS-CoV genome were previously predicted to fold into eight stem-loops (SL1 to SL8) [15]. To predict the RNA secondary structures at the 5′-proximal SARS-CoV-2 sequence and compare it to the 5′ ends of SARS-CoV and bat SARS-like CoV, the first 474 nts of the SARS-CoV-2 genomic sequence was analyzed using the Mfold web server. It should be noted that during the preparation of this manuscript, a recent study predicted conserved RNA structures within the SARS-CoV-2 genome, including RNA elements at the 5′ and 3′ ends [24]. The Mfold analysis predicted a SARS-CoV-2 5′-terminal RNA secondary structure model with eight RNA stem-loops (SL1–SL8) that was identical to the SARS-CoV model previously proposed by Yang *et al.* (Figure 4). In addition, the predicted model in this study was similar to that recently described in the bioinformatics study by Rangan *et al.*, except that seven stem-loops (SL1–SL7) were predicted. Herein, an additional stem-loop (SL8) was identified that was consistent with the SARS-CoV 5′ RNA structure model [15,24]. SL1, SL2 and SL4 are located within the 5′ UTRs of coronavirus genomes and they are structurally conserved among at least three coronavirus genera, with SL2 being the most conserved RNA secondary structure [25]. In addition, these results demonstrated that the conserved core leader of the transcriptional regulatory sequence (TRS-L) region required for subgenomic RNA synthesis is located within SL3, which is a finding consistent with previous studies of SARS-CoV and BCoV (Figure 4) [25]. The predicted SL4, which has been shown to be conserved in all CoVs, is longer than the three preceding stem-loops and contains a short upstream ORF that is found in most CoVs [13,25,26]. Moreover, a long stem-loop RNA structure that contains three hairpin substructures (termed as SL5A, 5B and 5C) was also identified (Figure 4) [14]. Part of the SL5 is located in the 5′ UTR; however, the AUG initiation codon of the nsp1 is located downstream of SL5C at a position similar with previous studies on SARS-CoV (Figure 4). It should be noted that SL5ABC is conserved among betacoronaviruses, suggesting essential roles, such as viral replication, of these stem-loops structures as CREs in the life cycle of CoVs. In agreement with a previous study on SARS-CoV, the loops of SL5A and SL5B contain the conserved 5′-UUUCGU-3′ motifs and this is equivalent to the conserved 5′-UUYCGU-3′ loop sequences found in SL5ABC of alphacoronaviruses [13,25]. Bioinformatics analysis predicted three stem-loops (SL6, SL7 and SL8) in the nsp1 coding sequence similar to that found in the 5′-terminal region of SARS-CoV (Figure 4). However, these RNA structures are known to be less conserved between CoV lineages than the stem-loops found within the 5′ UTR of CoV genomes.

**Figure 5.**
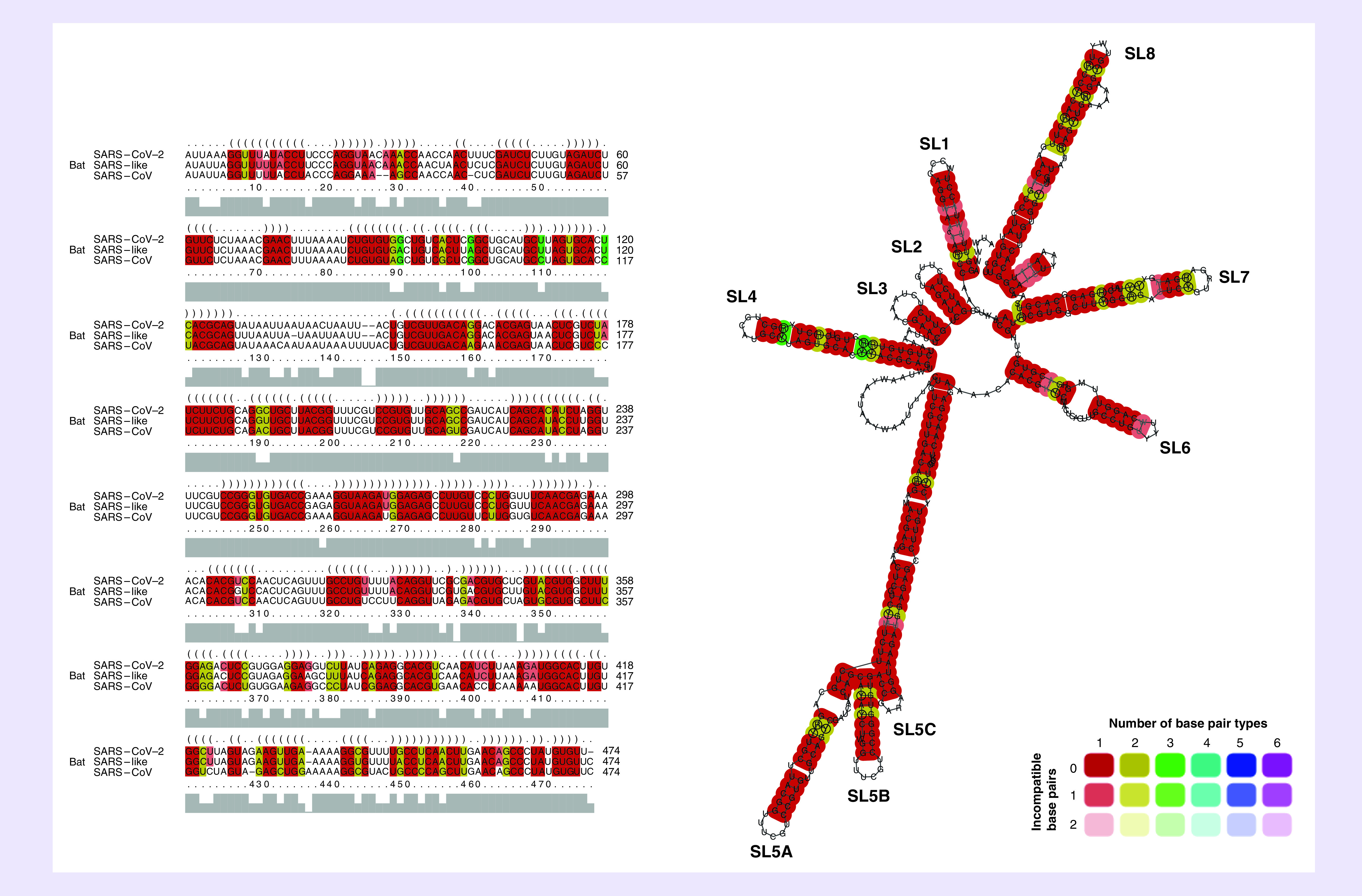
Alignment-based prediction of the RNA structures at the 5′-terminal regions of severe acute respiratory syndrome coronavirus 2 genomes. The first 474 nucleotides of SARS-CoV-2, bat SARS-like CoV and SARS-CoV genomes were aligned using LocARNA to predict conserved RNA stem-loops at the 5′ ends. Colors indicate the conservation of base pair types in which red represents conservation of one base pair and yellow and green indicate conservation of two and three base pairs, respectively. Dark color represents that all three viruses contained this base pair, whereas light color shows that one or two of the sequences did not contain the base pairs. SARS-CoV-2: Severe acute respiratory syndrome coronavirus 2.

To further confirm the conservation of the RNA secondary structures located at the 5′ regions among SARS-related viruses, the first 474 nts of SARS-CoV-2, SARS-CoV and bat SARS-like CoV genomes were aligned and folded using the LocARNA web server [18]. Sequence alignments indicated that all the RNA stem-loops were highly conserved among the three viruses, with SL2 and SL3 being the most conserved among the other stem-loops with sequence covariation (Figure 5). This high degree of conservation was expected for SL2 and SL3, because SL2 is suggested to be the most conserved RNA element in the CoV 5′ UTR region. SL3, which is found in SARS-like CoVs and BCoVs, contains TRS-L sequences that have an essential role in subgenomic RNA synthesis [13,27]. These results indicated high conservation of RNA elements at the 5′ ends of SARS-like CoV genomes, hence suggesting that these RNA secondary structures also function as CREs in the SARS-CoV-2 life cycle.

## Discussion

*Cis*-acting RNA elements have been described and characterized in several RNA virus genomes, including picornaviruses, coronaviruses and noroviruses [11,28,29]. These CREs play important functional roles in the virus life cycle and usually contribute to viral RNA replication, translation and genome packaging [11]. Previous studies have identified stem-loop RNA structures located at the 3′ end of the SARS-CoV ORF1b, which are recognized by the SARS-CoV structural N protein and suggested to act as the genomic PS [21,22]. Therefore, this work aimed to use bioinformatics tools to identify if the genome of the newly emerged SARS-CoV-2 shares conserved CREs present within the 5′ end and the ORF1ab regions of the viral genome.

Computational analysis predicted two conserved stem-loops within the 3′ end of the ORF1b region (named as SL1 and SL2), the latter of which have been previously described to be part of the SARS-CoV PS and was named as PS_core_ [22]. SL2 is more conserved among the three viruses than SL1 and the top of SL2 is featured with a hexaloop that contains a U-rich motif. It should be noted that genomic PSs for different CoVs consist of RNA structure elements that vary in length and genomic location within the same lineages [14,30]. For example, the genomic PS of MERS-CoV, which is a lineage C *Betacoronavirus*, was identified in a similar position as SARS-CoV at the 3′ end of the ORF1ab region [31]. Moreover, the genomic PS is functionally and structurally conserved in lineage A betacoronaviruses that contain a 95 nt stem-loop RNA structure and located within the 3′ region of ORF1b [32]. However, the genomic PS of transmissible gastroenteritis virus is located at the 5′-terminal end of the viral genome [33]. Although this study cannot conclude the functionality of the predicted RNA stem-loop structures located at the SARS-CoV-2 ORF1b region and their roles in the SARS-CoV-2 life cycle, it is postulated that they may similarly function as *cis*-acting elements. Hence, they could be a putative genomic PS for SARS-CoV-2 because of: the similar position of the viral genome where these RNA elements are located at the 3′ end of ORF1b; and and the conserved RNA secondary structures and sequences of these predicted stem-loops when compared with those found in the closely related SARS-CoV and bat SARS-like CoV.

This study also used genome sequences at the 5′-proximal region of the SARS-CoV-2 genome to predict conserved RNA secondary structures and compare to SARS-related CoVs, such as SARS-CoV and bat SARS-like CoV. The predicted model in this study was similar to a previous model described for SARS-CoV, in which eight RNA stem-loops (SL1–SL8) were identified [15]. In addition, the RNA structures at the 5′ region of SARS-CoV-2 were similar to structures recently described in a bioinformatics study; however, the only difference was that SL8 was not identified in the previous study [24]. The RNA secondary structures of SL1, SL2 and SL4 are conserved across the three CoV genera, whereas SL1, SL2 and SL4 and SL5ABC are only conserved among betacoronaviruses [15].

## Conclusion

In summary, this study used computational tools to predict *cis*-acting RNA motifs in the SARS-CoV-2 RNA genome. Bioinformatics analysis suggested that the 3′ end of the SARS-CoV-2 ORF1b region may contain RNA structure elements structurally conserved with other SARS-CoVs and analogous to the SARS-CoV genomic packaging signal. This study also demonstrated and confirmed predicted RNA secondary structures at the 5′-proximal region of the SARS-CoV-2 genome. However, further studies are warranted to: biochemically confirm these CREs; and to investigate their roles in the virus life cycle. These important regions within the SARS-CoV-2 genome could then be targeted to develop a vaccine and/or antiviral therapeutics.

**Summary points**

- Severe acute respiratory syndrome coronavirus 2 (SARS-CoV-2) is responsible for the ongoing global pandemic of coronavirus disease 2019.
- Similar to most RNA viruses, the SARS-CoV-2 genome contains *cis*-acting RNA elements that interact with RNA and viral or host protein.
- A previous bioinformatics study on the SARS-CoV genome predicted a stable stem-loop RNA structure at the 3′ end of ORF1b, to be the putative core PS (PScore).
- Bioinformatics analysis identified two stable stem-loops that are similar and observed in the 3′ end of three SARS-CoVs ORF1b region (including SARS-CoV-2).
- One of the identified stem-loop (SL2) was the previously predicted to function as the putative core PS (PScore) of SARS-CoV genome.
- Therefore, it is reasonable to assume that the predicted RNA stem-loop structures of SARS-CoV-2 may also have the same role as a putative genomic packaging signal.
- The Mfold analysis predicted a SARS-CoV-2 5′-terminal RNA secondary structure model that was identical to the SARS-CoV model previously proposed.
- Alignment-based prediction of the RNA structures at the 5′-terminal regions were highly conserved among three SARS-like CoVs.
- Further studies are warranted to: biochemically confirm these *cis*-acting RNA elements; and to investigate their roles in the virus life cycle.
